# Temporal Trends in Mortality Due to Ischemic Heart Disease and Cardiac Arrhythmias in the United States From 1999 to 2023

**DOI:** 10.1002/clc.70450

**Published:** 2026-08-14

**Authors:** Taha Alam, Sohaima Kamal, Dhvanit Rajdeep, F. N. U. Areesha, Pardeep Kumar, Hamza Yousuf Ibrahim, Muhammad Suleman Khan, Muhammad Ibrahim Rashid, Alisha Ahmed, Muhammad Raafay Jamil, Nicholas Aderinto, Ehsanullah Alokozay, Caleb Karani, Hassaan Abid

**Affiliations:** ^1^ Dow Medical College Dow University of Health Sciences Karachi Pakistan; ^2^ Department of Medicine Caucasus International University Tbilisi Georgia; ^3^ Department of Medicine Liaquat University of Medical and Health Sciences Jamshoro Sindh Pakistan; ^4^ Deparment of Medicine Jinnah Medical and Dental College Karachi Pakistan; ^5^ Department of Medicine Pakistan Naval Ship (PNS) Shifa Karachi Pakistan; ^6^ Department of Medicine Bahria University College of Medicine, Bahria University Islamabad Pakistan; ^7^ Department of Medicine Jinnah Sindh Medical University Karachi Pakistan; ^8^ Department of Medicine Quaid‐e‐Azam Medical College Bahawalpur Pakistan; ^9^ Department of Medicine Ladoke Akintola University of Technology Ogbomosho Nigeria; ^10^ Faculty of Medicine Kandahar University Kandahar Afghanistan; ^11^ Faculty of Health Sciences University of Nairobi Nairobi Kenya; ^12^ Department of Internal Medicine Indiana University School of Medicine Muncie Indiana USA

**Keywords:** arrhythmias, CDC wonders, ischemic heart disease, join point analysis

## Abstract

**Background:**

Ischemic heart disease (IHD) remains a leading cause of mortality in the United States, while arrhythmia‐related deaths are increasing. However, long‐term mortality trends involving both conditions remain unclear.

**Methods:**

We utilized the CDC WONDER Multiple Cause‐of‐Death database to obtain records from 1999 to 2023, targeting US adults aged ≥ 25 years. Deaths involving IHD and arrhythmias were assessed. Age‐adjusted mortality rates (AAMR) were calculated and stratified by gender, age, ethnicity, census region, and urbanization.

**Results:**

A total of 1 795 918 deaths were attributed to both IHD and cardiac arrhythmias between 1999 and 2023. The AAMR declined from 41.97 (95% CI: 41.66−42.27) in 1999 to 32.73 (95% CI: 32.51−32.94) in 2023, representing an overall decrease of 22% (AAPC −0.99). Men had higher AAMRs compared to women (45.0 [95% CI: 44.6−45.5] vs. 23.4 [95% CI: 23.2−23.7]). The highest mortality rate was observed among NH White individuals (AAMR: 34.8; 95% CI: 34.5−35.0), while the lowest was among NH Asian or Pacific Islanders (AAMR: 16.5; 95% CI: 15.6−17.5). Older adults exhibited the higher AAMRs (144.74; 95% CI: 143.60−145.88) compared to younger cohorts. An increased death burden was observed in non‐metropolitan (AAMR: 37.1; 95% CI: 36.5−37.8) and Midwest regions (35.5; 95% CI: 34.9−36.0). The majority of the deaths occurred in inpatient facilities (34.7%).

**Conclusions:**

Although IHD–arrhythmia mortality declined between 1999 and 2023, substantial disparities persisted across demographic and geographic subgroups, highlighting the need for targeted public health interventions.

## Graphical abstract

Mortality involving ischemic heart disease and cardiac arrhythmias declined over two decades before rising sharply during the COVID‐19 pandemic. Significant disparities by sex, race, region, and rurality emphasize the need for equitable cardiovascular prevention and management.

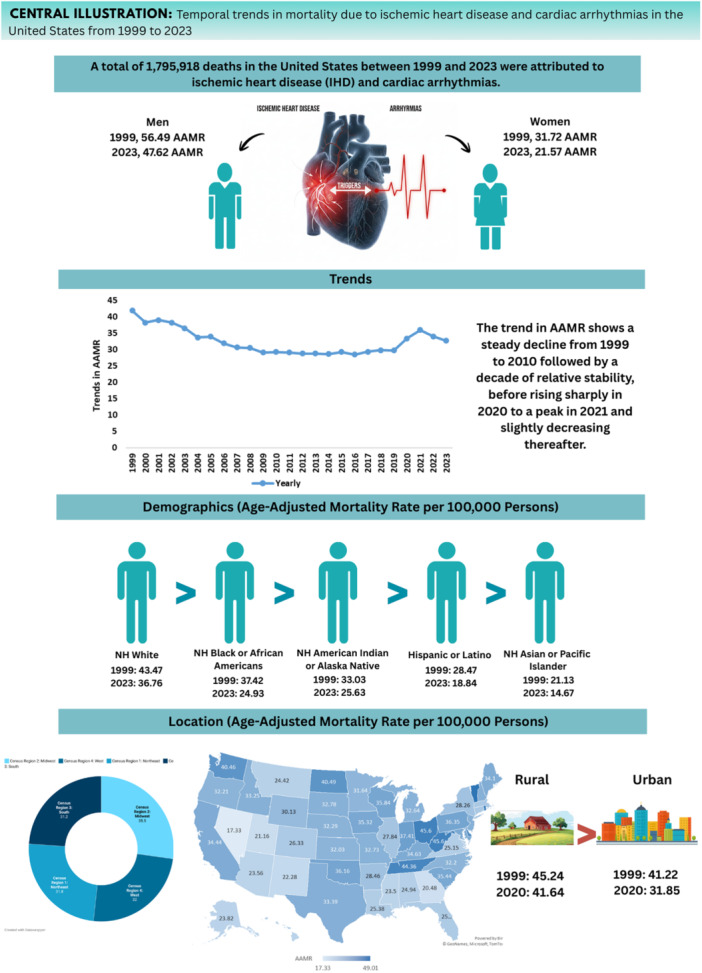

**Abbreviations**
AAMR: age‐adjusted mortality rate
APC: annual percent change
CDC WONDER: Centers for Disease Control and Prevention Wide‐Ranging Online Data for Epidemiological Research
CI: confidence interval
CMR: crude mortality rate
ICD‐10‐CM: International Classification of Diseases, Tenth Revision, Clinical Modification
IHD: ischemic heart disease
NH: non‐Hispanic

## 1 Introduction

Cardiovascular disease remains the leading cause of mortality, morbidity, and disability worldwide with ischemic heart disease (IHD) and stroke topping the charts [1, 2]. Disability‐adjusted life years (DALYs) is a parameter used to quantify the years of healthy life lost to disease, disability, or premature demise. The DALYs attributed to IHD have risen consistently since the 20th century reaching an astounding 182 million years globally [1]. According to recent American Heart Association data, IHD was responsible for 40% of US deaths in the year 2022, making it the leading cause of death by a huge mile in the country. The colossal disease burden directly costs the US government over 100 billion US dollars annually [3]. These findings signify that coronary artery disease persists as a major healthcare concern, especially among low socioeconomic index countries, men, and older individuals, highlighting gaps in healthcare access and preventive efforts [4, 5].

Similarly, the global and national burden of arrhythmias and associated mortality has continued to mount in the last two decades [6]. In the United States alone, over five million people experience cardiac arrhythmias annually, costing up to 67 billion USD in medical costs [7]. In the background of IHD, cardiac arrhythmias can have a variety of manifestations including atrial fibrillation, atrioventricular nodal blocks, and ventricular tachyarrhythmias, each reflecting the location, severity, and timing of myocardial damage [8, 9]. Ventricular tachycardia and ventricular fibrillation are particularly implicated post‐acute myocardial infarction [10, 11], and are increasingly recognized as terminal arrhythmias culminating in sudden cardiac deaths in young adults [12, 13]. On the other hand, studies have demonstrated that mortality among IHD patients, associated particularly with atrial fibrillation is on the rise compared to other arrhythmias [14, 15]. These emerging patterns, coupled with disparities in arrhythmia care stemming from differences in expert training, medical equipment, and advanced infrastructure across regions, call for a comprehensive review of combined effect of both diseases [16].

A complex and reciprocal relationship between myocardial ischemia and arrhythmias exists, where the presence of one leads to the other. Cardiac ischemia promotes ATP depletion, ionic imbalance, myocardial fibrosis, and electrical remodeling, creating conduction heterogeneity that predisposes to atrial and ventricular arrhythmias. Conversely, persistent arrhythmias can increase myocardial oxygen demand, shorten diastolic coronary perfusion, reduce cardiac output, and exacerbate myocardial oxygen supply‐demand mismatch, thereby worsening ischemia and perpetuating a vicious cycle [11, 17]. Although the overall IHD‐associated age‐adjusted mortality rates (AAMR) have decreased since the inception of the 21st century, IHD still remains the leading cause of death in the United States [18, 19]. Previous studies have largely evaluated IHD and cardiac arrhythmias separately or have focused on specific arrhythmia subtypes and selected patient populations. However, comprehensive national analyses examining long‐term mortality trends from the coexistence of IHD and cardiac arrhythmias across demographic and geographic subgroups remain limited [20, 21]. The CDC WONDER database is an extensive, publicly available repository of national death certificates that provides a wide range of epidemiological data for large‐scale analysis and is representative of the entire US population. We aim to analyze the collective national mortality trends of these two fatal diseases stratified according to age, gender, race, and geographical location for better prevention, management, and intervention. By analyzing CDC records, our study will assess whether IHD and arrhythmia‐related mortality rates have risen, declined, or remained stable in recent years and provide an estimate of the overall efficacy of current management practices, which will drive national healthcare reforms and optimize long‐term policy planning.

## 2 Materials and Methods

### 2.1 Study Setting and Population

This retrospective population‐based nationwide study used mortality data in the form of death certificates assembled by the CDC Wide‐Ranging Online Data for Epidemiologic Research (CDC WONDER) database. We analyzed mortality trends associated with IHD and arrhythmias targeting the US population aged 25 years and older, from the year 1999 to 2023. The Multiple Cause‐of‐Death (MCD) Public Use records were examined for mortality data, including evidence from all 50 states and the District of Columbia. Deaths were included when both IHD (ICD‐10 I20–I25) and cardiac arrhythmia (ICD‐10 I44, I45, I47–I49) codes were present anywhere on the death certificate, irrespective of whether either condition was listed as the underlying cause of death or as a contributing cause [14, 20, 21]. Therefore, our analysis reflects mortality involving the coexistence of IHD and cardiac arrhythmias rather than deaths attributable to one condition causing the other. According to CDC WONDER guidelines, instances with less than 20 mortalities per stratum were tagged as unreliable and were omitted from subsequent analysis. This study utilized anonymized, publicly shared data; thus, it was not subjected to institutional review board (IRB) approval. Transparency and systematic reporting were ensured by following the STROBE (Strengthening the Reporting of Observational Studies in Epidemiology) guidelines [22].

### 2.2 Data Extraction

Three authors were involved in data abstraction and verification. One independently extracted the following parameters: year of death, demographics (including age, gender, and ethnicity), and geographical information (including state, Census, metropolitan/non‐metropolitan). The other two authors verified the extracted data.

Mortality data for individuals aged 25 years and older were extracted and included in the analysis. Age groups were classified as: younger adults (25–44), middle‐aged adults (45–64), and older adults (65−85+). Race/ethnicity was classified into: Non‐Hispanic (NH) Black or African American, NH American Indian or Alaska Native, NH White, Hispanic or Latino, and NH Asian or Pacific Islander. Geographical data, including the metropolitan/non‐metropolitan classification, was based on the National Center for Health Statistics Urban–Rural Classification Scheme. For simplification of analysis, the six groups were consolidated into two broader categories: “Metropolitan” (comprising small metro, medium metro, large fringe metro, and large central metro areas) and “non‐metropolitan” (including non‐core rural and micropolitan areas) [23].

Metropolitan/non‐metropolitan data were only available for the years 1999–2020 and were abstracted from the Final MCD database. Due to the unavailability of grouping fields, MCD data for 2021–2023 were not used for metropolitan/non‐metropolitan analysis.

### 2.3 Statistical Analysis

We calculated the crude mortality rates (CMRs) and AAMRs per 100 000 population for each year. Age adjustment was done by standardizing the IHD and arrhythmia‐related deaths to the year 2000 US standard population [24]. CMR was calculated by dividing IHD and arrhythmia‐related deaths by the corresponding US population of that year. Rates were stratified by year, gender, age, ethnicity, and metropolitan/non‐metropolitan classification, with 95% confidence intervals (CIs) provided. To evaluate long‐term trends, we applied Joinpoint Regression Analysis (version V 5.4.0.0) developed by the National Cancer Institute [25]. The annual percent change (APC) with 95% CIs was estimated by fitting the log‐linear model to age‐adjusted rates over time. The Bayesian Information Criterion (BIC) was used to estimate the number and placement of join points. APCs were deemed as increasing or decreasing if the slope representing the change in mortality rate was significantly different from zero using two‐tailed *t*‐testing. Statistical significance was considered at *p* < 0.05. Subgroups with suppressed data were excluded from the Joinpoint analysis to maintain statistical integrity and model convergence. All analyses were conducted in accordance with the CDC WONDER documentation and best practices for trend analysis.

## 3 Results

A total of 1 795 918 deaths in the United States between 1999 and 2023 were attributed to IHD and cardiac arrhythmias (Table 1).

**Table 1:** Absolute number of deaths stratified by sex and race for IHD and arrhythmias in the United States, 1999–2023.

Year	Overall	Male	Female	NH Black or African American	NH White	American Indian or Alaska Native	Asian or Pacific Islander	Hispanic or Latino
1999	73 844	38 597	35 247	5467	64 946	227	780	2262
2000	68 265	35 565	32 700	4894	60 199	216	693	2077
2001	70 880	36 951	33 929	5170	61 898	214	914	2530
2002	70 472	36 736	33 736	5105	61 530	213	1009	2449
2003	68 399	35 780	32 619	5069	59 568	240	961	2448
2004	64 103	33 865	30 238	4785	55 632	243	907	2452
2005	65 813	34 700	31 113	4777	57 129	206	971	2639
2006	63 204	33 589	29 615	4600	54 799	257	1015	2463
2007	62 129	33 283	28 846	4444	54 190	218	922	2299
2008	63 120	33 751	29 369	4389	54 811	224	1080	2514
2009	61 308	33 393	27 915	4263	53 160	269	1053	2490
2010	62 584	34 476	28 108	4236	54 253	266	1122	2619
2011	64 203	35 427	28 776	4355	55 649	271	1179	2665
2012	65 068	36 287	28 781	4546	56 112	303	1236	2738
2013	66 836	37 812	29 024	4683	57 448	273	1324	2989
2014	67 896	38 920	28 976	4846	58 141	307	1320	3093
2015	70 925	40 962	29 963	4953	60 626	339	1432	3347
2016	70 618	41 322	29 296	5013	59 997	365	1521	3547
2017	74 334	44 041	30 293	5234	63 101	377	1686	3788
2018	77 271	46 098	31 173	5472	65 419	362	1805	4047
2019	78 867	47 434	31 433	5606	66 675	362	1850	4255
2020	90 043	54 803	35 240	6700	75 158	498	2166	5363
2021	93 162	57 325	35 837	6641	77 980	444	2205	5384
2022	93 134	57 228	35 906	6588	78 169	425	2253	5192
2023	89 440	55 368	34 072	6498	74 856	437	2157	4927
Total	1 795 918	1 013 713	782 205	128 334	1 541 446	7556	33 561	80 577

### 3.1 Annual Trends for IHD and Arrhythmias Related AAMRs

**Figure 1:**
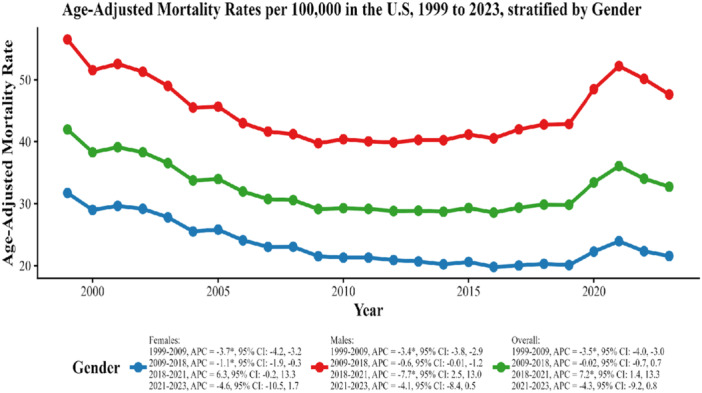
Sex‐stratified age‐adjusted mortality rates per 100 000 for IHD‐arrhythmias‐associated mortality in United States adults aged ≥ 25 years, 1999−2023. *Indicates that the annual percentage change (APC) is significantly different from zero at *α* = 0.05. APC = annual percentage change, CI = confidence interval.

The AAMR for IHD and arrhythmia‐related deaths declined from 41.97 in 1999 to 32.73 in 2023, representing an overall reduction of 22% during the study period. The AAMR declined significantly from 1999 to 2009 (APC: −3.50; 95% CI: −3.96 to −3.05), remained stable between 2009 and 2018 (APC: −0.02; 95% CI: −0.70 to 0.65), increased significantly from 2018 to 2021 (APC: +7.17; 95% CI: +1.41 to +13.26), and finally culminated in a nonsignificant decline thereafter between 2021 and 2023 (APC: −4.31; 95% CI: −9.17 to +0.80). The overall average annual percent change (AAPC) for the full study period was −0.99 (95% CI: −1.78 to −0.20), indicating a statistically significant long‐term decline in IHD and arrhythmia‐related mortality in the United States (Figure 1, Tables 1 and 2, and Supporting Information S1: Table 1).

### 3.2 IHD and Arrhythmias Related AAMRs Stratified by Sex

Men exhibited persistently higher mortality rates than women throughout the study period. In 1999, AAMR was 56.49 for men and 31.72 for women, declining to 47.62 and 21.57, respectively, in 2023. Joinpoint analysis demonstrated a significant decline among men from 1999 to 2009 (APC: −3.36; 95% CI: −3.80 to −2.92), followed by a stable period between 2009 and 2018 (APC: +0.59; 95% CI: −0.01 to +1.20), a significant increase between 2018 and 2021 (APC: +7.65; 95% CI: +2.55 to +13.00), and a slight decline thereafter (2021–2023, APC: −4.06; 95% CI: −8.40 to +0.49). Among women, AAMR declined significantly from 1999 to 2009 (APC: −3.68; 95% CI: −4.18 to −3.18) and continued to decline significantly from 2009 to 2018 (APC: −1.11; 95% CI: −1.86 to −0.34), followed by a nonsignificant rise between 2018 and 2021 (APC: +6.31; 95% CI: −0.22 to +13.26) and a drop thereafter (2021–2023, APC: −4.62; 95% CI: −10.51 to +1.67) (Figure 1, Tables 1 and 2, and Supporting Information S1: Table 1).

### 3.3 IHD and Arrhythmias Related AAMRs Stratified by Race

The AAMRs for IHD‐ and arrhythmia‐related deaths showed an overall downward trend across all racial and ethnic groups from 1999 to 2023, though the magnitude of decline varied substantially. The highest AAMRs were observed among NH White individuals, followed by NH Black or African American, NH American Indian or Alaska Native, Hispanic, and NH Asian or Pacific Islander populations.

For NH White individuals, the AAMR declined significantly from 1999 to 2009 (APC: −3.23; 95% CI: −3.68 to −2.79), followed by a period of stability from 2009 to 2018 (APC: +0.33; 95% CI: −0.32 to +0.98), a significant increase from 2018 to 2021 (APC: +7.61; 95% CI: +1.95 to +13.59), and a modest decline between 2021 and 2023 (APC: −3.63; 95% CI: −8.67 to +1.69).

Among NH Black or African American individuals, the AAMR declined significantly from 1999 to 2010 (APC: −4.22; 95% CI: −4.75 to −3.68), followed by a stable period between 2010 and 2018 (APC: −0.58; 95% CI: −1.68 to +0.53), a nonsignificant increase from 2018 to 2021 (APC: +6.39; 95% CI: −1.26 to +14.63), and a numerical decline between 2021 and 2023 (APC: −3.65; 95% CI: −10.29 to +3.49).

In NH American Indian or Alaska Native individuals, the AAMR showed a slight downward trend from 1999 to 2023 (APC: −0.29; 95% CI: −0.82 to +0.25) without statistical significance. The overall AAPC was −0.29 (95% CI: −0.82 to +0.25; nonsignificant), indicating a relatively stable trend in mortality over time.

For NH Asian or Pacific Islander individuals, the AAMR decreased significantly from 1999 to 2014 (APC: −2.77; 95% CI: −3.64 to −1.91), followed by a significant increase from 2014 to 2023 (APC: +1.48; 95% CI: +0.06 to +2.91).

**Figure 2:**
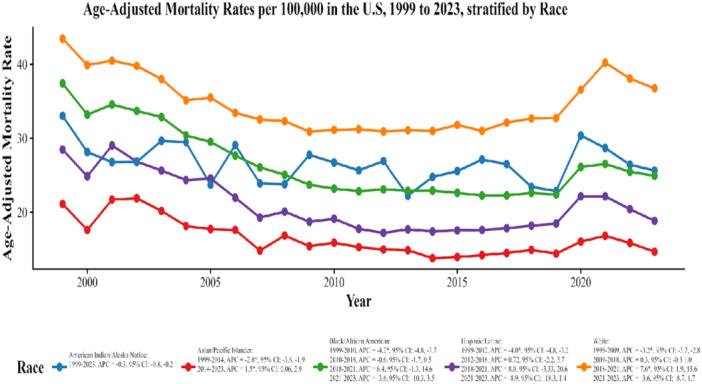
Race‐stratified age‐adjusted mortality rates per 100 000 for IHD‐arrythmias‐associated mortality in United States adults aged ≥ 25 years, 1999−2023. *Indicates that the annual percentage change (APC) is significantly different from zero at *α* = 0.05. APC = annual percentage change, CI = confidence interval.

Among Hispanic individuals, the AAMR declined significantly from 1999 to 2012 (APC: −4.01; 95% CI: −4.79 to −3.22), remained stable between 2012 and 2018 (APC: +0.72; 95% CI: −2.20 to +3.73), increased moderately from 2018 to 2021 (APC: +7.97; 95% CI: −3.33 to +20.58), and declined again between 2021 and 2023 (APC: −8.94; 95% CI: −18.27 to +1.45) (Figure 2, Tables 1 and 2, and Supporting Information S1: Table 2).

### 3.4 IHD and Arrhythmias Related AAMRs Stratified by Age

**Figure 3:**
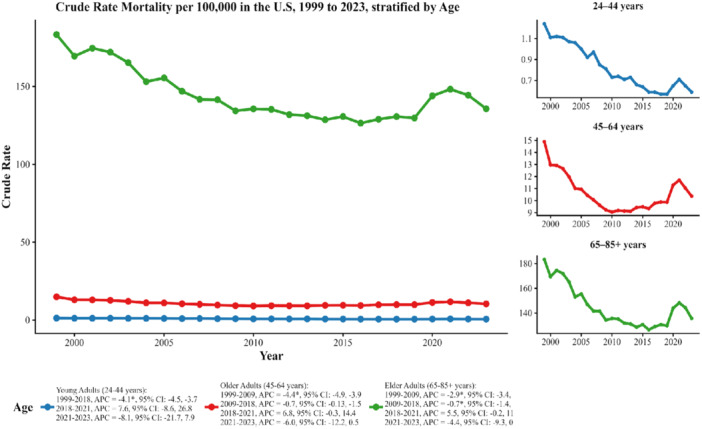
Age‐stratified crude mortality rates per 100 000 for IHD‐arrhythmias‐associated mortality in United States adults aged ≥ 25 years, 1999−2023. *Indicates that the annual percentage change (APC) is significantly different from zero at *α* = 0.05.). APC = annual percentage change, CI = confidence interval.

Age‐stratified analysis revealed the highest mortality among older adults (≥ 65 years), followed by middle‐aged adults (45–64 years) and younger adults (25–44 years). Between 1999 and 2023, the CMR declined across all age groups. Among young adults, the CMR decreased from 1.24 in 1999 to 0.59 in 2023, with a significant decline observed from 1999 to 2018 (APC: −4.10; 95% CI: −4.48 to −3.72), followed by a nonsignificant increase between 2018 and 2021 (APC: +7.62; 95% CI: −8.64 to +26.77) and a subsequent decline from 2021 to 2023 (APC: −8.09; 95% CI: −21.74 to +7.93). Similarly, among adults aged 45–64 years, the CMR declined from 14.89 in 1999 to 10.37 in 2023, with a significant reduction between 1999 and 2009 (APC: −4.41; 95% CI: −4.92 to −3.89). Finally, among older adults, the CMR declined from 183.33 in 1999 to 135.66 in 2023, with a significant decrease between 1999 and 2009 (APC: −2.94; 95% CI: −3.41 to −2.46), followed by a significant decline from 2009 to 2018 (APC: −0.71; 95% CI: −1.38 to −0.04), a nonsignificant increase during 2018–2021 (APC: +5.53; 95% CI: −0.16 to +11.54), and a nonsignificant decline between 2021 and 2023 (APC: −4.40; 95% CI: −9.33 to +0.80) (Figure 3, Table 2, and Supporting Information S1: Table 3).

**Table 2:** Annual percent change (APC) and average annual percent change (AAPC) in age‐adjusted mortality rates (per 100 000) for IHD and arrhythmias in the United States, 1999–2023.

Year segment	APC (95% CI)	*p* value	AAPC (95% CI)	*p* value
Overall				
1999−2009	−3.5033* (−3.9558 to −3.0485)	< 0.000001	−0.9916* (−1.7813 to −0.1955)	0.01473
2009−2018	−0.0244 (−0.6979 to 0.6536)	0.93927		
2018−2021	7.1737* (1.4135 to 13.2612)	0.017608		
2021−2023	−4.314 (−9.1707 to 0.8025)	0.090891		
Sex				
Female				
1999−2009	−3.6830* (−4.1814 to −3.1819)	< 0.000001	−1.5965* (−2.509 to −0.6754)	0.00071
2009−2018	−1.1052* (−1.8637 to −0.3409)	0.007891		
2018−2021	6.3083 (−0.2154 to 13.2585)	0.057243		
2021−2023	−4.615 (−10.5103 to 1.6686)	0.134508		
Male				
1999−2009	−3.3589* (−3.7963 to −2.9195)	< 0.000001	−0.6241 (−1.3276 to 0.0845)	0.084174
2009−2018	0.5917 (−0.0144 to 1.2015)	0.054962		
2018−2021	7.6504* (2.5498 to 13.0047)	0.005729		
2021−2023	−4.0578 (−8.4042 to 0.4949)	0.075961		
Race				
American Indian or Alaska Native				
1999−2023	−0.288 (−0.8226 to 0.2495)	0.278503	−0.288 (−0.8226 to 0.2495)	0.278503
Asian or Pacific Islander				
1999−2014	−2.7747* (−3.6367 to −1.905)	0.000002	−1.2021* (−1.9114 to −0.4877)	0.001002
2014−2023	1.4755* (0.0581 to 2.913)	0.042042		
Black or African American				
1999−2010	−4.2207* (−4.7549 to −3.6835)	< 0.000001	−1.6913* (−2.7589 to −0.6119)	0.002201
2010−2018	−0.579 (−1.6803 to 0.5346)	0.282344		
2018−2021	6.3865 (−1.2608 to 14.6261)	0.096785		
2021−2023	−3.646 (−10.2922 to 3.4925)	0.283792		
White				
1999−2009	−3.2343* (−3.679 to −2.7876)	< 0.000001	−0.6346 (−1.4197 to 0.1567)	0.115709
2009−2018	0.3323 (−0.3153 to 0.9841)	0.290398		
2018−2021	7.6120* (1.9452 to 13.5938)	0.011445		
2021−2023	−3.6305 (−8.6717 to 1.689)	0.162026		
Hispanic or Latino				
1999−2012	−4.0089* (−4.787 to −3.2245)	< 0.000001	−1.8420* (−3.4953 to −0.1604)	0.031937
2012−2018	0.7205 (−2.2026 to 3.731)	0.609253		
2018−2021	7.966 (−3.33 to 20.5819)	0.159056		
2021−2023	−8.9411 (−18.2654 to 1.447)	0.08409		
Urbanization				
Metropolitan				
1999−2009	−3.6354* (−4.1115 to −3.1569)	< 0.000001	−1.2546* (−1.8534 to −0.6522)	0.000047
2009−2018	−0.1459 (−0.8591 to 0.5725)	0.668945		
2018−2020	6.0941* (0.2328 to 12.2981)	0.042425		
Non‐metropolitan				
1999−2009	−2.8191* (−3.3049 to −2.3308)	< 0.000001	−0.4112 (−0.9434 to 0.1239)	0.131775
2009−2017	0.4137 (−0.4597 to 1.2946)	0.327996		
2017−2020	5.7035* (2.6433 to 8.8549)	0.001194		
Census region				
Census region 1: Northeast				
1999−2007	−4.1822* (−5.011 to −3.3462)	< 0.000001	−1.5597* (−2.671 to −0.4357)	0.00665
2007−2018	−0.9104* (−1.5541 to −0.2624)	0.009375		
2018−2021	5.9163 (−2.054 to 14.5351)	0.137392		
2021−2023	−5.2256 (−12.2684 to 2.3826)	0.158209		
Census region 2: Midwest				
1999−2010	−3.0148* (−3.4147 to −2.6133)	< 0.000001	−0.9303* (−1.756 to −0.0978)	0.028591
2010−2018	0.2715 (−0.5685 to 1.1186)	0.500721		
2018−2021	7.2796* (1.3172 to 13.5928)	0.01957		
2021−2023	−5.8278* (−10.9045 to −0.4619)	0.035689		
Census region 3: South				
1999−2009	−3.8096* (−4.3656 to −3.2503)	< 0.000001	−0.7167 (−1.6179 to 0.1926)	0.122036
2009−2018	0.2537 (−0.5507 to 1.0646)	0.510908		
2018−2021	8.9684* (2.3746 to 15.987)	0.010521		
2021−2023	−3.1835 (−8.6789 to 2.6426)	0.254805		
Census region 4: West				
1999−2009	−3.1502* (−3.7473 to −2.5494)	< 0.000001	−0.9884 (−1.9718 to 0.0048)	0.051112
2009−2018	0.0371 (−0.7803 to 0.8612)	0.924172		
2018−2021	5.3338 (−1.6646 to 12.8302)	0.127293		
2021−2023	−3.803 (−9.8575 to 2.6582)	0.221617		
Age				
Young adults (25−44 years)				
1999−2018	−4.0993* (−4.4799 to −3.7172)	< 0.000001	−3.0517* (−5.2481 to −0.8043)	0.008033
2018−2021	7.6172 (−8.6435 to 26.7723)	0.357653		
2021−2023	−8.0917 (−21.7354 to 7.9304)	0.283361		
Older adults (45−64 years)				
1999−2009	−4.4088* (−4.9248 to −3.8901)	< 0.000001	−1.3161* (−2.2981 to −0.3243)	0.009416
2009−2018	0.6788 (−0.1316 to 1.4958)	0.094221		
2018−2021	6.7806 (−0.3035 to 14.368)	0.059599		
2021−2023	−6.0479 (−12.2007 to 0.5361)	0.068268		
Elder adults (65+ years)				
1999−2009	−2.9350* (−3.4082 to −2.4595)	< 0.000001	−1.2052* (−1.9993 to −0.4047)	0.003232
2009−2018	−0.7140* (−1.3819 to −0.0416)	0.039017		
2018−2021	5.5271 (−0.1603 to 11.5384)	0.056099		
2021−2023	−4.4015 (−9.3335 to 0.7989)	0.089787		

### 3.5 IHD and Arrhythmias Related AAMRs Stratified by Urbanization

**Figure 4:**
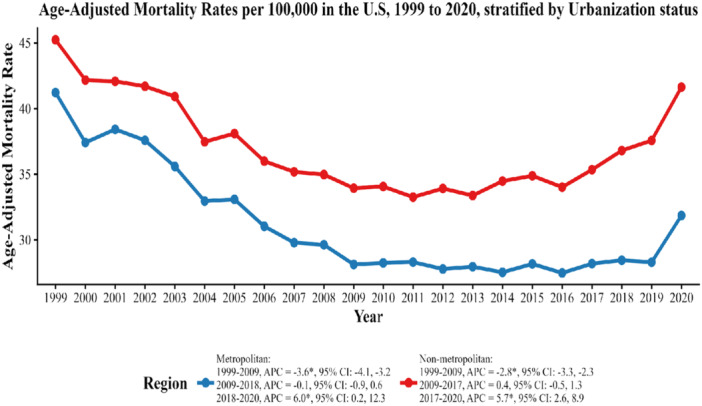
Urbanization stratified age‐adjusted mortality rates per 100 000 for IHD‐arrhythmias‐associated mortality in United States adults aged ≥ 25 years, 1999−2020. *Indicates that the annual percentage change (APC) is significantly different from zero at *α* = 0.05. APC = annual percentage change, CI = confidence interval.

Non‐metropolitan areas experienced consistently higher mortality than metropolitan regions (overall mean AAMR: 37.1 vs. 31.2). In 1999, AAMRs were 45.24 in non‐metropolitan and 41.22 in metropolitan regions, declining to 41.64 and 31.85, respectively, by 2020. Joinpoint regression revealed a significant decline in metropolitan areas from 1999 to 2009 (APC: −3.64; 95% CI: −4.11 to −3.16) followed by steady trend until 2018 and a significant increase thereafter (2018–2020, APC: +6.09; 95% CI: +0.23 to +12.30). Non‐metropolitan areas exhibited a significant early decline (1999–2009, APC: −2.82; 95% CI: −3.30 to −2.33), steady change from 2009 to 2017, and a significant rise 2017–2020 (APC: +5.70; 95% CI: +2.64 to +8.85), suggesting a widening rural–urban mortality gap in recent years (Figure 4, Table 2, and Supporting Information S1: Table 4).

### 3.6 IHD and Arrhythmias Related AAMRs Stratified by Census Region

Over the course of the study, the highest mortality was observed in the Midwestern region, followed by the Western, Southern, and Northeastern regions.

For the Northeast, the AAMR declined significantly from 1999 to 2007 (APC: −4.18; 95% CI: −5.01 to −3.35), followed by a continued but slower decline from 2007 to 2018 (APC: −0.91; 95% CI: −1.55 to −0.26). The rate then showed a mild, nonsignificant rise between 2018 and 2021 (APC: +5.92; 95% CI: −2.05 to +14.54), before decreasing again between 2021 and 2023 (APC: −5.23; 95% CI: −12.27 to +2.38).

In the Midwest, mortality declined significantly from 1999 to 2010 (APC: −3.01; 95% CI: −3.41 to −2.61), followed by a stable phase between 2010 and 2018 (APC: +0.27; 95% CI: −0.57 to +1.12). A significant increase was observed from 2018 to 2021 (APC: +7.28; 95% CI: +1.32 to +13.59), followed by a significant decline between 2021 and 2023 (APC: −5.83; 95% CI: −10.90 to −0.46).

For the South, the AAMR declined significantly from 1999 to 2009 (APC: −3.81; 95% CI: −4.37 to −3.25), followed by a period of stability from 2009 to 2018 (APC: +0.25; 95% CI: −0.55 to +1.06), a significant increase from 2018 to 2021 (APC: +8.97; 95% CI: +2.37 to +15.99), and a numerical decline from 2021 to 2023 (APC: −3.18; 95% CI: −8.68 to +2.64).

**Figure 5:**
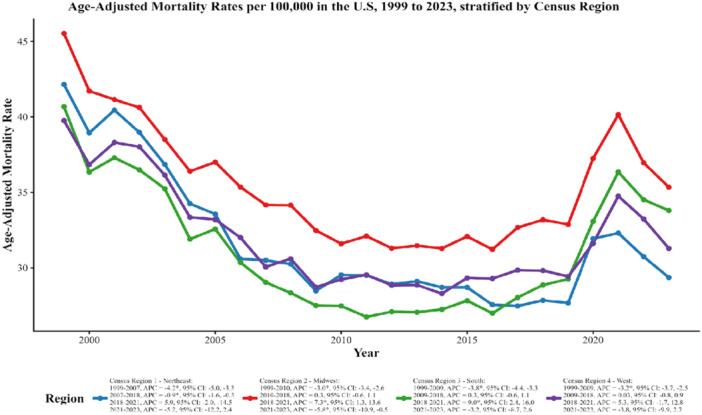
Census region stratified age‐adjusted mortality rates per 100 000 for IHD‐arrhythmias‐associated mortality in United States adults aged ≥ 25 years, 1999−2020. *Indicates that the annual percentage change (APC) is significantly different from zero at *α* = 0.05. APC = annual percentage change, CI = confidence interval.

In the West, the AAMR declined significantly from 1999 to 2009 (APC: −3.15; 95% CI: −3.75 to −2.55), followed by a stable phase from 2009 to 2018 (APC: +0.04; 95% CI: −0.78 to +0.86). A nonsignificant increase occurred between 2018 and 2021 (APC: +5.33; 95% CI: −1.66 to +12.83), followed by a nonsignificant decline from 2021 to 2023 (APC: −3.80; 95% CI: −9.86 to +2.66) (Figure 5, Table 2, and Supporting Information S1: Table 5).

### 3.7 IHD and Arrhythmias Related AAMRs Stratified by State

AAMRs ranged from 20.0 in Georgia to 48.4 in Vermont. States in the top 90th percentile (Vermont, West Virginia, Tennessee, Ohio, Rhode Island, Wisconsin) exhibited nearly double the mortality burden of those in the lowest 10th percentile (Georgia, Nevada, Hawaii, Connecticut, Utah, Alaska), highlighting the continued influence of regional healthcare disparities and demographic risk factors (Supporting Information S1: Figures 1 and 2 and Tables 6 and 7).

### 3.8 IHD and Arrhythmias Related AAMRs Stratified by Place of Death

Among all deaths, 34.7% occurred within inpatient facilities, 25.0% at home, 21.1% in nursing homes or long‐term care facilities, 11.5% in outpatient or emergency settings, 3.0% in hospice, 3.6% in other locations, and 1.0% were categorized as dead‐on arrival (Supporting Information S1: Figure 3 and Table 8).

## 4 Discussion

Between 1999 and 2023, the United States recorded 1 795 918 deaths attributed to concomitant IHD and cardiac arrhythmias. Our CDC WONDER analysis documents a 22% decline in AAMRs during this period, though substantial heterogeneity persists across demographic and geographic subgroups. Because this investigation employs an ecological design examining aggregated population data, findings describe temporal associations and demographic patterns without establishing causal relationships [22, 26].

The period from 1999 to 2009 witnessed the steepest mortality decline (APC: −3.50). This steep decline corresponds with the expanded adoption of guideline‐directed medical therapies, including statins and antihypertensives, alongside improved population‐level control of traditional risk factors [27, 28]. Furthermore, advances in acute coronary syndrome management, including greater risk of primary percutaneous coronary intervention and evidence‐based secondary prevention strategies, as well as improvements in arrhythmia management like implantable cardioverter‐defibrillators for prevention of sudden cardiac death, may have contributed to the observed decline in mortality [29]. Our population‐level mortality data, however, lack the granularity to quantify how much each therapeutic advance contributed to the observed decline. The mortality trend flattened substantially between 2009 and 2017 (APC: −0.02), mirroring patterns seen in other national data sets where cardiovascular gains slowed markedly after 2011 [30, 31].

Mortality rates reversed course sharply during 2017 to 2021 (APC: +7.17), a period overlapping with the COVID‐19 pandemic. Swedish registry studies documented that SARS‐CoV‐2 infection increased the risk of myocardial infarction, IHD, heart failure, and arrhythmias for more than a year after the acute illness had resolved [32]. This reversal likely stems from a synergistic combination of direct viral cardiotoxicity and widespread healthcare disruptions, including canceled elective procedures and avoidance of emergency care [33, 34]. Hospitalized COVID‐19 patients died from cardiovascular causes at four times the rate of matched individuals without infection [35].

Men died at consistently higher rates than women across the entire 25‐year span. Male age‐adjusted mortality stood at 47.62 per 100 000 in 2023, while female mortality reached only 21.57 per 100 000. Biology alone, however, explains only part of the sex gap. Women frequently experience delays in diagnosis and receive less aggressive treatment, partly due to atypical symptom presentation and systemic under‐prescription of guideline‐recommended therapies [36, 37]. Younger women under 60 who undergo percutaneous coronary intervention face higher complication rates and greater in‐hospital mortality than their male counterparts, according to pooled analyses [38].

Racial and ethnic inequalities remained entrenched throughout the observation period. NH White populations recorded the highest mean AAMRs, though their overall trend showed no statistical significance (AAPC: −0.63; 95% CI: −1.42 to +0.16). NH Black populations achieved meaningful long‐term mortality reductions (AAPC: −1.69; 95% CI: −2.76 to −0.61) yet still suffered disproportionately high cardiovascular death rates relative to other groups [39]. Current consensus attributes racial health gaps to the structural environment rather than biological susceptibility [40]. Residential segregation and underinvestment in community infrastructure concentrate cardiovascular risk in minority populations by limiting access to preventive resources [41].

Data from the 2022 National Health Interview Survey linked rural−urban mortality differences to social hardship more than to any other measured variable. Poverty, food insecurity, incomplete high school education, and unstable housing accounted for most of the difference between regions. Healthcare access and lifestyle choices, by contrast, explained little of the inequality [42]. Black and Hispanic populations experience higher rates of housing and food insecurity, lower insurance coverage, and reduced access to mental health services, all amplifying cardiovascular risk [43].

Mortality rates tracked closely with age and reached their highest levels among adults older than 65 years. Aging remains the potent driver of mortality, correlating with progressive vascular dysfunction, oxidative stress, accumulation of traditional risk factors and an increasing burden of comorbidities [44]. Age related degeneration of the cardiac conduction system and myocardial fibrosis also increase susceptibility to clinically significant arrhythmias, further contributing to the elevated mortality observed in older adults. Frailty, characterized by multisystem physiological decline, correlates with increased arrhythmia symptom severity and worse cardiovascular outcomes independent of chronological age [45].

The rural−urban mortality gap widened throughout the study period, especially after 2017. Non‐metropolitan areas maintained consistently higher AAMRs (37.1 vs. 31.2 per 100 000) than metropolitan regions. *JAMA Cardiology* reported that poverty and joblessness tracked more closely with rural mortality gaps than clinic availability [42]. Hospital shutdowns and uneven Medicaid coverage in rural states likely intensified this pattern [42]. Limited access to specialized cardiovascular services, including electrophysiology care and PCI‐capable centers, may also contribute to poorer outcomes among rural populations. During the COVID‐19 pandemic, rural populations, particularly NH Black and American Indian or Alaska Native communities, experienced disproportionate mortality increases linked with lower vaccination rates and underfunded healthcare infrastructure [46].

The Midwest bore the heaviest mortality toll among all four census regions examined. State‐level breakdown exposed a striking geographic divide, with Georgia at 20.0 deaths per 100 000 and Vermont at 48.4 per 100 000 [47]. Behind this 2.4‐fold variation stand multiple overlapping factors. Medicaid expansion divided the country along state lines. Some states embraced the policy while others rejected it outright. Cigarette smoking and obesity clustered differently across regions. Wages and educational completion rates swung dramatically from state to state. Vermont's substantially older population certainly contributed to its elevated rates. However, aging alone fails to explain why some states suffered so much more than others. Economic hardship, healthcare access barriers, and population health infrastructure vary dramatically across state lines and account for much of the geographic inequality we observed. Our findings on arrhythmia deaths parallel what CDC surveillance has revealed. Arrhythmia mortality climbed from 111.4 per 100 000 in 1999 to 137.3 by 2023, suggesting a genuine epidemiological shift in how these cardiac conditions account for premature death. Regional differences in the implementation of evidence based cardiovascular prevention and treatment strategies may also contribute to these persistent geographic disparities [48].

### 4.1 Clinical Implications

The stark disparities we documented across different demographic groups and regions point to three urgent research gaps. We need longitudinal studies that follow individuals over time and collect detailed information on factors that could be changed to improve health outcomes and explain why population mortality trends have shifted [49]. Implementation science must test how to get proven cardiovascular treatments to patients in rural and medically underserved areas [49]. Natural experiments that exploit state policy differences, particularly states that did and did not expand Medicaid or reformed how they pay rural hospitals, can reveal which interventions actually work to close geographic health gaps [42]. The cardiovascular toll of COVID‐19 exposed how unprepared the healthcare system remains for major public health crises. Better surveillance that tracks both virus spread and cardiovascular events simultaneously could alert us sooner to cardiovascular complications emerging during future pandemics [50]. Upstream interventions addressing root causes of poor health, such as food banks, affordable housing programs, and better schools, deserve serious investment and evaluation [40, 51]. When researchers co‐design solutions with the communities they serve, the resulting programs tend to fit better and work more effectively [41].

### 4.2 Strengths and Limitations

Our analysis spans 25 years through 2023, capturing mortality trends across the full COVID‐19 period. CDC WONDER aggregates death certificate data from all 50 states and US territories, providing population‐level counts with near‐complete coverage. We acknowledge limitations inherent to administrative database research. Relying on death certificates introduces potential misclassification bias, as physicians sometimes code the immediate mechanism of death (cardiac arrest) rather than the underlying condition that triggered it. We could not access information on treatment timing, specific devices used in revascularization, or other clinical details that would sharpen our risk adjustment. Urbanization classification stopped being updated after 2020, preventing analysis of how rural versus metropolitan mortality shifted during the acute pandemic period (2020−2021) and the recovery that followed. Additionally, population‐level data allows us to identify associations but not to establish causality between IHD and arrhythmias in individual patients.

## 5 Conclusion

From 1999 through 2023, mortality from IHD and arrhythmias declined overall but reversed during the COVID‐19 pandemic. Disparities across sex, race, age, and geography tell a more complete story. Men suffer excess mortality compared with women across the lifespan. Older adults, rural populations, and racial and ethnic minority groups experienced disproportionately higher mortality. These patterns correlate closely with structural inequities in healthcare access, economic opportunity, and residential environments Prospective longitudinal cohorts that collect individual risk factor data would illuminate which specific exposures drive the observed population trends. Quasi‐experimental studies of state policy variation, particularly Medicaid expansion decisions, could identify healthcare interventions that reduce geographic inequities.

## Author Contributions

**Taha Alam:** conceptualization, data curation, formal analysis, supervision, investigation, methodology, project administration, writing–original draft, writing– review and editing, visualization. **Dhvanit Rajdeep:** formal analysis, investigation, writing–original draft, writing–review and editing. **F. N. U. Areesha:** investigation, validation, methodology, writing–original draft. **Sohaima Kamal:** formal analysis, data curation, project administration, writing–review and editing. **Pardeep Kumar:** formal analysis, data curation, writing–original draft. **Muhammad Suleman Khan:** investigation, methodology, writing–original draft, writing–review and editing. **Muhammad Ibrahim Rashid:** investigation, methodology, writing–original draft. **Alisha Ahmed:** supervision, investigation, validation, writing–review and editing. **Muhammad Raafay Jamil:** investigation, project administration, writing–original draft, writing–review and editing. **Nicholas Aderinto:** validation, investigation, writing–review and editing. **Hamza Yousuf Ibrahim:** validation, project administration, writing–review and editing.

## Funding

The authors have nothing to report.

## Ethics Statement

The research is based on de‐identified data and does not require the consent of the participants or ethical approval.

## Conflicts of Interest

The authors declare no conflicts of interest.

## Supporting information


Supporting File

## Data Availability

The data sets generated and/or analyzed during the current study are available from the corresponding author on reasonable request.
