# Supplementary material for: Temporal Trends in Mortality Due to Ischemic Heart Disease and Cardiac Arrhythmias in the United States From 1999 to 2023
Source: Clin Cardiol. 2026 Aug 14;49(8):e70450. doi: 10.1002/clc.70450 (PMC13475013; doi:10.1002/clc.70450)
Supplement: Supplementary file 1 [file CLC-49-e70450-s001.docx]

**Supplementary Material**

**Temporal trends in mortality due to ischemic heart disease and cardiac arrhythmias in the United States from 1999 to 2023**

**TABLES**

**Supplemental Table 1:** Overall and sex-stratified age-adjusted mortality rates per 100,000 for ischemic heart disease (IHD) and arrythmias in the United States, 1999–2023.

| **Year** | **Overall** | **Male** | **Female** |
| --- | --- | --- | --- |
| **1999** | 41.97 (41.66 - 42.27) | 56.49 (55.92 - 57.07) | 31.72 (31.39 - 32.05) |
| **2000** | 38.29 (38.00 - 38.58) | 51.54 (50.99 - 52.08) | 28.99 (28.67 - 29.30) |
| **2001** | 39.10 (38.81 - 39.38) | 52.55 (52.01 - 53.10) | 29.65 (29.34 - 29.97) |
| **2002** | 38.30 (38.01 - 38.58) | 51.29 (50.75 - 51.82) | 29.16 (28.84 - 29.47) |
| **2003** | 36.54 (36.27 - 36.82) | 48.98 (48.46 - 49.49) | 27.80 (27.50 - 28.11) |
| **2004** | 33.75 (33.49 - 34.01) | 45.51 (45.02 - 46.01) | 25.52 (25.23 - 25.81) |
| **2005** | 33.97 (33.71 - 34.23) | 45.65 (45.16 - 46.14) | 25.81 (25.52 - 26.10) |
| **2006** | 31.95 (31.70 - 32.20) | 43.00 (42.53 - 43.46) | 24.10 (23.83 - 24.38) |
| **2007** | 30.73 (30.49 - 30.97) | 41.63 (41.17 - 42.08) | 23.03 (22.77 - 23.30) |
| **2008** | 30.57 (30.34 - 30.81) | 41.21 (40.76 - 41.65) | 23.05 (22.78 - 23.32) |
| **2009** | 29.13 (28.90 - 29.36) | 39.75 (39.32 - 40.18) | 21.53 (21.27 - 21.78) |
| **2010** | 29.27 (29.04 - 29.50) | 40.39 (39.96 - 40.82) | 21.31 (21.06 - 21.57) |
| **2011** | 29.15 (28.92 - 29.37) | 40.05 (39.63 - 40.47) | 21.31 (21.06 - 21.56) |
| **2012** | 28.81 (28.59 - 29.04) | 39.88 (39.46 - 40.29) | 20.91 (20.66 - 21.15) |
| **2013** | 28.86 (28.64 - 29.08) | 40.28 (39.87 - 40.70) | 20.69 (20.45 - 20.94) |
| **2014** | 28.70 (28.48 - 28.92) | 40.24 (39.83 - 40.64) | 20.22 (19.99 - 20.46) |
| **2015** | 29.30 (29.09 - 29.52) | 41.16 (40.76 - 41.57) | 20.61 (20.37 - 20.85) |
| **2016** | 28.55 (28.34 - 28.77) | 40.54 (40.14 - 40.93) | 19.81 (19.58 - 20.04) |
| **2017** | 29.37 (29.16 - 29.59) | 41.99 (41.59 - 42.39) | 20.04 (19.81 - 20.27) |
| **2018** | 29.84 (29.63 - 30.05) | 42.77 (42.37 - 43.16) | 20.30 (20.08 - 20.53) |
| **2019** | 29.81 (29.60 - 30.02) | 42.85 (42.46 - 43.24) | 20.11 (19.89 - 20.34) |
| **2020** | 33.42 (33.20 - 33.64) | 48.48 (48.07 - 48.89) | 22.27 (22.04 - 22.51) |
| **2021** | 36.06 (35.83 - 36.30) | 52.21 (51.78 - 52.65) | 23.95 (23.70 - 24.20) |
| **2022** | 34.04 (33.82 - 34.26) | 50.14 (49.73 - 50.56) | 22.33 (22.10 - 22.56) |
| **2023** | 32.73 (32.51 - 32.94) | 47.62 (47.21 - 48.02) | 21.57 (21.34 - 21.80) |
| **Total** | 32.49 (32.25 - 32.73) | 45.0 (44.6 - 45.5) | 23.4 (23.2 - 23.7) |

**Supplemental Table 2:** Race-stratified age-adjusted mortality rates per 100,000 for ischemic heart disease (IHD) and arrythmias in the United States, 1999–2023.

| **Year** | **Hispanic/Latino** | **NH American Indian or Alaska Native** | **NH Asian or Pacific Islander** | **NH Black or African American** | **NH White** |
| --- | --- | --- | --- | --- | --- |
| **1999** | 28.47 (27.25 - 29.70) | 33.03 (28.49 - 37.57) | 21.13 (19.57 - 22.68) | 37.42 (36.42 - 38.42) | 43.47 (43.14 - 43.81) |
| **2000** | 24.86 (23.75 - 25.98) | 28.14 (24.19 - 32.09) | 17.61 (16.24 - 18.97) | 33.20 (32.26 - 34.14) | 39.90 (39.58 - 40.22) |
| **2001** | 29.03 (27.86 - 30.21) | 26.78 (22.99 - 30.56) | 21.71 (20.25 - 23.17) | 34.56 (33.60 - 35.51) | 40.49 (40.17 - 40.81) |
| **2002** | 26.85 (25.75 - 27.96) | 26.80 (22.98 - 30.62) | 21.90 (20.50 - 23.31) | 33.70 (32.76 - 34.64) | 39.79 (39.48 - 40.11) |
| **2003** | 25.64 (24.59 - 26.70) | 29.66 (25.68 - 33.65) | 20.18 (18.85 - 21.50) | 32.87 (31.95 - 33.79) | 38.01 (37.71 - 38.32) |
| **2004** | 24.35 (23.35 - 25.35) | 29.48 (25.56 - 33.40) | 18.14 (16.92 - 19.35) | 30.39 (29.51 - 31.27) | 35.13 (34.84 - 35.42) |
| **2005** | 24.58 (23.61 - 25.55) | 23.72 (20.25 - 27.19) | 17.73 (16.58 - 18.88) | 29.52 (28.67 - 30.37) | 35.49 (35.20 - 35.78) |
| **2006** | 21.98 (21.08 - 22.88) | 29.09 (25.30 - 32.87) | 17.62 (16.50 - 18.73) | 27.67 (26.85 - 28.49) | 33.44 (33.16 - 33.72) |
| **2007** | 19.28 (18.46 - 20.09) | 23.90 (20.52 - 27.28) | 14.83 (13.84 - 15.81) | 26.07 (25.29 - 26.86) | 32.54 (32.26 - 32.81) |
| **2008** | 20.09 (19.28 - 20.90) | 23.80 (20.48 - 27.11) | 16.86 (15.83 - 17.89) | 25.07 (24.31 - 25.82) | 32.32 (32.05 - 32.60) |
| **2009** | 18.72 (17.96 - 19.48) | 27.76 (24.24 - 31.27) | 15.42 (14.46 - 16.37) | 23.72 (22.99 - 24.45) | 30.90 (30.63 - 31.16) |
| **2010** | 19.12 (18.36 - 19.87) | 26.72 (23.32 - 30.12) | 15.88 (14.94 - 16.83) | 23.20 (22.49 - 23.92) | 31.13 (30.87 - 31.39) |
| **2011** | 17.76 (17.07 - 18.45) | 25.66 (22.43 - 28.90) | 15.29 (14.40 - 16.18) | 22.84 (22.15 - 23.54) | 31.23 (30.97 - 31.49) |
| **2012** | 17.22 (16.55 - 17.88) | 26.92 (23.73 - 30.12) | 14.97 (14.13 - 15.82) | 23.09 (22.40 - 23.78) | 30.92 (30.66 - 31.17) |
| **2013** | 17.69 (17.04 - 18.34) | 22.25 (19.46 - 25.04) | 14.87 (14.05 - 15.68) | 22.90 (22.23 - 23.57) | 31.08 (30.82 - 31.34) |
| **2014** | 17.41 (16.79 - 18.04) | 24.77 (21.88 - 27.67) | 13.80 (13.05 - 14.56) | 22.93 (22.27 - 23.59) | 30.98 (30.72 - 31.23) |
| **2015** | 17.55 (16.94 - 18.15) | 25.56 (22.71 - 28.40) | 13.94 (13.21 - 14.67) | 22.62 (21.98 - 23.27) | 31.81 (31.55 - 32.07) |
| **2016** | 17.61 (17.02 - 18.21) | 27.12 (24.23 - 30.01) | 14.21 (13.48 - 14.93) | 22.26 (21.63 - 22.90) | 31.00 (30.75 - 31.25) |
| **2017** | 17.84 (17.26 - 18.43) | 26.51 (23.74 - 29.28) | 14.50 (13.80 - 15.20) | 22.27 (21.65 - 22.89) | 32.12 (31.87 - 32.38) |
| **2018** | 18.19 (17.61 - 18.76) | 23.44 (20.94 - 25.93) | 14.92 (14.23 - 15.62) | 22.63 (22.01 - 23.24) | 32.67 (32.42 - 32.92) |
| **2019** | 18.47 (17.90 - 19.03) | 22.86 (20.43 - 25.28) | 14.41 (13.75 - 15.08) | 22.39 (21.79 - 22.99) | 32.73 (32.48 - 32.98) |
| **2020** | 22.16 (21.55 - 22.77) | 30.37 (27.63 - 33.11) | 16.04 (15.36 - 16.73) | 26.12 (25.48 - 26.76) | 36.57 (36.31 - 36.84) |
| **2021** | 22.16 (21.55 - 22.77) | 28.69 (25.94 - 31.44) | 16.82 (16.12 - 17.53) | 26.53 (25.87 - 27.18) | 40.23 (39.94 - 40.51) |
| **2022** | 20.42 (19.85 - 20.99) | 26.43 (23.86 - 28.99) | 15.85 (15.19 - 16.51) | 25.48 (24.85 - 26.11) | 38.07 (37.80 - 38.34) |
| **2023** | 18.84 (18.30 - 19.38) | 25.63 (23.18 - 28.08) | 14.67 (14.05 - 15.29) | 24.93 (24.30 - 25.55) | 36.76 (36.49 - 37.03) |
| **Total** | 21.1 (20.3 - 21.8) | 26.6 (23.4 - 29.8) | 16.5 (15.6 - 17.5) | 26.6 (25.8 - 27.3) | 34.8 (34.5 - 35.0) |

**Supplemental Table 3:** Age group-stratified crude mortality rates per 100,000 for ischemic heart disease (IHD) and arrythmias in the United States, 1999–2023.

| **Crude Mortality Rates** | | | |
| --- | --- | --- | --- |
| **Year** | **Young Adults (25-44 years)** | **Older Adults (45-64 years)** | **Elder Adults (65+ years)** |
| **1999** | 1.24 (1.17 - 1.32) | 14.89 (14.59 - 15.20) | 183.33 (181.91 - 184.75) |
| **2000** | 1.11 (1.04 - 1.18) | 12.97 (12.68 - 13.25) | 169.43 (168.06 - 170.79) |
| **2001** | 1.12 (1.05 - 1.19) | 12.92 (12.64 - 13.20) | 174.57 (173.19 - 175.94) |
| **2002** | 1.11 (1.04 - 1.18) | 12.66 (12.39 - 12.93) | 171.99 (170.62 - 173.35) |
| **2003** | 1.07 (1.00 - 1.14) | 11.98 (11.72 - 12.24) | 165.25 (163.92 - 166.58) |
| **2004** | 1.06 (0.99 - 1.13) | 11.02 (10.77 - 11.26) | 153.05 (151.78 - 154.32) |
| **2005** | 1.00 (0.93 - 1.07) | 10.94 (10.70 - 11.18) | 155.48 (154.20 - 156.75) |
| **2006** | 0.92 (0.86 - 0.99) | 10.44 (10.21 - 10.67) | 146.89 (145.66 - 148.12) |
| **2007** | 0.97 (0.90 - 1.03) | 10.06 (9.84 - 10.29) | 141.64 (140.44 - 142.84) |
| **2008** | 0.85 (0.79 - 0.91) | 9.62 (9.40 - 9.84) | 141.46 (140.28 - 142.64) |
| **2009** | 0.81 (0.75 - 0.87) | 9.23 (9.02 - 9.44) | 134.35 (133.20 - 135.49) |
| **2010** | 0.73 (0.67 - 0.79) | 9.06 (8.85 - 9.27) | 135.60 (134.46 - 136.73) |
| **2011** | 0.74 (0.68 - 0.80) | 9.19 (8.99 - 9.40) | 135.24 (134.12 - 136.36) |
| **2012** | 0.71 (0.66 - 0.77) | 9.15 (8.94 - 9.35) | 131.88 (130.79 - 132.96) |
| **2013** | 0.73 (0.67 - 0.78) | 9.12 (8.92 - 9.33) | 131.20 (130.13 - 132.26) |
| **2014** | 0.66 (0.60 - 0.71) | 9.44 (9.23 - 9.65) | 128.58 (127.55 - 129.62) |
| **2015** | 0.64 (0.59 - 0.70) | 9.49 (9.28 - 9.70) | 130.66 (129.63 - 131.68) |
| **2016** | 0.59 (0.54 - 0.64) | 9.34 (9.13 - 9.54) | 126.40 (125.41 - 127.40) |
| **2017** | 0.59 (0.54 - 0.64) | 9.78 (9.57 - 10.00) | 128.93 (127.94 - 129.91) |
| **2018** | 0.57 (0.52 - 0.62) | 9.89 (9.67 - 10.10) | 130.61 (129.63 - 131.59) |
| **2019** | 0.57 (0.52 - 0.62) | 9.88 (9.67 - 10.10) | 129.74 (128.78 - 130.70) |
| **2020** | 0.65 (0.60 - 0.70) | 11.29 (11.06 - 11.52) | 143.96 (142.96 - 144.95) |
| **2021** | 0.71 (0.65 - 0.76) | 11.70 (11.47 - 11.93) | 148.19 (147.18 - 149.20) |
| **2022** | 0.65 (0.59 - 0.70) | 11.04 (10.82 - 11.27) | 144.38 (143.40 - 145.36) |
| **2023** | 0.59 (0.54 - 0.64) | 10.37 (10.15 - 10.59) | 135.66 (134.72 - 136.60) |
| **Total** | 0.82 (0.76 - 0.88) | 10.62 (10.39 - 10.85) | 144.74 (143.60 - 145.88) |

**Supplemental Table 4:** Urbanization-stratified age-adjusted mortality rates per 100,000 for ischemic heart disease (IHD) and arrythmias in the United States, 1999–2023.

| **Age-Adjusted Mortality Rates** | | |
| --- | --- | --- |
| **Year** | **Metropolitan** | **Non-metropolitan** |
| **1999** | 41.22 (40.89 - 41.56) | 45.24 (44.52 - 45.96) |
| **2000** | 37.41 (37.09 - 37.72) | 42.18 (41.49 - 42.88) |
| **2001** | 38.42 (38.11 - 38.74) | 42.08 (41.39 - 42.77) |
| **2002** | 37.58 (37.27 - 37.89) | 41.70 (41.02 - 42.38) |
| **2003** | 35.59 (35.29 - 35.89) | 40.92 (40.25 - 41.59) |
| **2004** | 32.95 (32.66 - 33.24) | 37.47 (36.83 - 38.11) |
| **2005** | 33.08 (32.79 - 33.36) | 38.10 (37.45 - 38.74) |
| **2006** | 31.03 (30.76 - 31.30) | 35.99 (35.37 - 36.61) |
| **2007** | 29.78 (29.52 - 30.05) | 35.18 (34.57 - 35.79) |
| **2008** | 29.62 (29.36 - 29.89) | 34.97 (34.37 - 35.57) |
| **2009** | 28.12 (27.87 - 28.38) | 33.93 (33.34 - 34.52) |
| **2010** | 28.24 (27.99 - 28.49) | 34.06 (33.47 - 34.66) |
| **2011** | 28.30 (28.05 - 28.55) | 33.25 (32.67 - 33.83) |
| **2012** | 27.77 (27.53 - 28.01) | 33.91 (33.33 - 34.49) |
| **2013** | 27.95 (27.71 - 28.19) | 33.37 (32.80 - 33.94) |
| **2014** | 27.52 (27.29 - 27.75) | 34.47 (33.89 - 35.05) |
| **2015** | 28.16 (27.92 - 28.39) | 34.88 (34.31 - 35.46) |
| **2016** | 27.47 (27.24 - 27.69) | 34.01 (33.45 - 34.58) |
| **2017** | 28.18 (27.95 - 28.41) | 35.34 (34.77 - 35.92) |
| **2018** | 28.44 (28.21 - 28.67) | 36.80 (36.22 - 37.38) |
| **2019** | 28.29 (28.06 - 28.51) | 37.57 (36.99 - 38.15) |
| **2020** | 31.85 (31.61 - 32.09) | 41.64 (41.04 - 42.25) |
| **Total** | 31.2 (31.0 - 31.5) | 37.1 (36.5 - 37.8) |

**Supplemental Table 5:** Census-stratified age-adjusted mortality rates per 100,000 for ischemic heart disease (IHD) and arrythmias in the United States, 1999–2023.

| **Age-Adjusted Mortality Rates** | | | | |
| --- | --- | --- | --- | --- |
| **Year** | **Census Region 1: Northeast** | **Census Region 2: Midwest** | **Census Region 3: South** | **Census Region 4: West** |
| **1999** | 42.15 (41.49 - 42.80) | 45.53 (44.89 - 46.17) | 40.68 (40.18 - 41.19) | 39.75 (39.09 - 40.42) |
| **2000** | 38.94 (38.32 - 39.57) | 41.71 (41.10 - 42.32) | 36.35 (35.88 - 36.82) | 36.84 (36.21 - 37.48) |
| **2001** | 40.46 (39.82 - 41.09) | 41.15 (40.55 - 41.76) | 37.30 (36.82 - 37.78) | 38.31 (37.67 - 38.95) |
| **2002** | 38.97 (38.35 - 39.59) | 40.62 (40.02 - 41.21) | 36.49 (36.02 - 36.96) | 38.02 (37.39 - 38.65) |
| **2003** | 36.86 (36.26 - 37.46) | 38.51 (37.93 - 39.09) | 35.25 (34.79 - 35.71) | 36.15 (35.55 - 36.76) |
| **2004** | 34.25 (33.68 - 34.83) | 36.40 (35.84 - 36.96) | 31.90 (31.47 - 32.33) | 33.35 (32.77 - 33.92) |
| **2005** | 33.56 (32.99 - 34.12) | 37.00 (36.45 - 37.56) | 32.57 (32.14 - 33.00) | 33.20 (32.63 - 33.76) |
| **2006** | 30.59 (30.06 - 31.13) | 35.35 (34.81 - 35.90) | 30.35 (29.94 - 30.76) | 32.00 (31.45 - 32.55) |
| **2007** | 30.50 (29.96 - 31.03) | 34.17 (33.64 - 34.70) | 29.04 (28.64 - 29.43) | 30.06 (29.53 - 30.58) |
| **2008** | 30.25 (29.72 - 30.77) | 34.15 (33.62 - 34.67) | 28.34 (27.95 - 28.73) | 30.60 (30.07 - 31.12) |
| **2009** | 28.48 (27.97 - 28.99) | 32.48 (31.97 - 32.99) | 27.51 (27.13 - 27.89) | 28.72 (28.22 - 29.23) |
| **2010** | 29.53 (29.01 - 30.04) | 31.60 (31.10 - 32.10) | 27.48 (27.11 - 27.86) | 29.23 (28.72 - 29.73) |
| **2011** | 29.50 (28.99 - 30.01) | 32.10 (31.60 - 32.60) | 26.76 (26.40 - 27.12) | 29.54 (29.04 - 30.03) |
| **2012** | 28.92 (28.41 - 29.42) | 31.30 (30.81 - 31.79) | 27.10 (26.74 - 27.46) | 28.84 (28.35 - 29.32) |
| **2013** | 29.11 (28.61 - 29.61) | 31.47 (30.98 - 31.95) | 27.07 (26.72 - 27.43) | 28.86 (28.38 - 29.33) |
| **2014** | 28.71 (28.21 - 29.20) | 31.29 (30.80 - 31.77) | 27.25 (26.90 - 27.61) | 28.30 (27.84 - 28.77) |
| **2015** | 28.71 (28.22 - 29.20) | 32.08 (31.59 - 32.56) | 27.83 (27.48 - 28.18) | 29.33 (28.87 - 29.80) |
| **2016** | 27.56 (27.08 - 28.04) | 31.22 (30.74 - 31.70) | 27.00 (26.66 - 27.34) | 29.29 (28.83 - 29.75) |
| **2017** | 27.48 (27.00 - 27.95) | 32.67 (32.18 - 33.15) | 28.04 (27.70 - 28.39) | 29.85 (29.39 - 30.31) |
| **2018** | 27.85 (27.38 - 28.32) | 33.18 (32.70 - 33.66) | 28.86 (28.51 - 29.20) | 29.82 (29.37 - 30.27) |
| **2019** | 27.68 (27.21 - 28.14) | 32.88 (32.40 - 33.35) | 29.26 (28.92 - 29.61) | 29.43 (28.98 - 29.87) |
| **2020** | 31.93 (31.43 - 32.43) | 37.25 (36.75 - 37.76) | 33.07 (32.71 - 33.43) | 31.62 (31.17 - 32.07) |
| **2021** | 32.30 (31.79 - 32.81) | 40.15 (39.61 - 40.68) | 36.35 (35.97 - 36.74) | 34.75 (34.26 - 35.23) |
| **2022** | 30.74 (30.26 - 31.23) | 36.96 (36.46 - 37.46) | 34.52 (34.16 - 34.89) | 33.24 (32.78 - 33.70) |
| **2023** | 29.36 (28.89 - 29.83) | 35.34 (34.85 - 35.83) | 33.80 (33.44 - 34.16) | 31.29 (30.84 - 31.74) |
| **Total** | 31.8 (31.2 - 32.3) | 35.5 (34.9 - 36.0) | 31.2 (30.8 - 31.6) | 32.0 (31.5 - 32.5) |

**Supplemental Table 6:** State-stratified age-adjusted mortality rates per 100,000 for ischemic heart disease (IHD) and arrythmias in the United States, 1999–2023.

| **Age-Adjusted Mortality Rates** | | | |
| --- | --- | --- | --- |
| **State** | **1999-2020** | **2021-2023** | **1999-2023** |
| **Alabama** | 24.94 (24.58 - 25.31) | 24.65 (23.76 - 25.53) | 24.8 (24.2 - 25.4) |
| **Alaska** | 23.82 (22.52 - 25.12) | 25.35 (22.42 - 28.28) | 24.6 (22.5 - 26.7) |
| **Arizona** | 23.56 (23.26 - 23.87) | 32.48 (31.67 - 33.29) | 28.0 (27.5 - 28.6) |
| **Arkansas** | 28.46 (27.98 - 28.95) | 40.30 (38.85 - 41.76) | 34.4 (33.4 - 35.4) |
| **California** | 34.44 (34.27 - 34.60) | 29.77 (29.41 - 30.13) | 32.1 (31.8 - 32.4) |
| **Colorado** | 26.33 (25.92 - 26.73) | 40.07 (38.92 - 41.22) | 33.2 (32.4 - 34.0) |
| **Connecticut** | 26.49 (26.09 - 26.89) | 19.58 (18.69 - 20.46) | 23.0 (22.4 - 23.7) |
| **Delaware** | 41.83 (40.77 - 42.89) | 39.58 (37.20 - 41.96) | 40.7 (39.0 - 42.4) |
| **District of Columbia** | 25.65 (24.57 - 26.73) | 25.24 (22.49 - 27.98) | 25.4 (23.5 - 27.4) |
| **Florida** | 25.96 (25.80 - 26.12) | 31.04 (30.62 - 31.46) | 28.5 (28.2 - 28.8) |
| **Georgia** | 20.48 (20.22 - 20.74) | 19.52 (18.93 - 20.11) | 20.0 (19.6 - 20.4) |
| **Hawaii** | 23.73 (23.11 - 24.36) | 20.74 (19.37 - 22.11) | 22.2 (21.2 - 23.2) |
| **Idaho** | 33.25 (32.48 - 34.01) | 40.35 (38.41 - 42.29) | 36.8 (35.4 - 38.2) |
| **Illinois** | 27.84 (27.61 - 28.08) | 24.51 (23.96 - 25.07) | 26.2 (25.8 - 26.6) |
| **Indiana** | 37.41 (37.02 - 37.79) | 40.65 (39.65 - 41.65) | 39.0 (38.3 - 39.7) |
| **Iowa** | 35.32 (34.83 - 35.81) | 41.27 (39.88 - 42.66) | 38.3 (37.4 - 39.2) |
| **Kansas** | 32.03 (31.51 - 32.55) | 38.01 (36.56 - 39.45) | 35.0 (34.0 - 36.0) |
| **Kentucky** | 34.63 (34.17 - 35.08) | 39.77 (38.56 - 40.98) | 37.2 (36.4 - 38.0) |
| **Louisiana** | 25.38 (24.99 - 25.77) | 36.18 (35.02 - 37.35) | 30.8 (30.0 - 31.6) |
| **Maine** | 34.10 (33.37 - 34.83) | 42.63 (40.63 - 44.62) | 38.4 (37.0 - 39.7) |
| **Maryland** | 35.16 (34.76 - 35.57) | 39.72 (38.69 - 40.75) | 37.4 (36.7 - 38.2) |
| **Massachusetts** | 26.77 (26.47 - 27.08) | 28.26 (27.48 - 29.05) | 27.5 (27.0 - 28.1) |
| **Michigan** | 32.64 (32.36 - 32.92) | 32.79 (32.07 - 33.50) | 32.7 (32.2 - 33.2) |
| **Minnesota** | 31.64 (31.26 - 32.02) | 46.51 (45.37 - 47.64) | 39.1 (38.3 - 39.8) |
| **Mississippi** | 23.50 (23.04 - 23.96) | 39.53 (38.03 - 41.04) | 31.5 (30.5 - 32.5) |
| **Missouri** | 32.73 (32.37 - 33.09) | 29.07 (28.21 - 29.92) | 30.9 (30.3 - 31.5) |
| **Montana** | 24.42 (23.68 - 25.17) | 33.90 (31.76 - 36.04) | 29.2 (27.7 - 30.6) |
| **Nebraska** | 32.29 (31.65 - 32.93) | 47.22 (45.23 - 49.20) | 39.8 (38.4 - 41.1) |
| **Nevada** | 17.33 (16.88 - 17.78) | 26.63 (25.39 - 27.86) | 22.0 (21.1 - 22.8) |
| **New Hampshire** | 33.08 (32.30 - 33.85) | 32.03 (30.21 - 33.86) | 32.6 (31.3 - 33.9) |
| **New Jersey** | 33.25 (32.95 - 33.55) | 27.57 (26.89 - 28.24) | 30.4 (29.9 - 30.9) |
| **New Mexico** | 22.28 (21.75 - 22.82) | 27.48 (26.06 - 28.89) | 24.9 (23.9 - 25.9) |
| **New York** | 28.26 (28.08 - 28.45) | 29.37 (28.90 - 29.83) | 28.8 (28.5 - 29.1) |
| **North Carolina** | 32.20 (31.90 - 32.50) | 35.82 (35.07 - 36.57) | 34.0 (33.5 - 34.5) |
| **North Dakota** | 40.49 (39.36 - 41.62) | 36.34 (33.58 - 39.10) | 38.4 (36.5 - 40.4) |
| **Ohio** | 45.60 (45.30 - 45.90) | 43.88 (43.12 - 44.64) | 44.7 (44.2 - 45.3) |
| **Oklahoma** | 36.16 (35.66 - 36.65) | 45.75 (44.35 - 47.15) | 41.0 (40.0 - 41.9) |
| **Oregon** | 32.21 (31.76 - 32.65) | 49.92 (48.57 - 51.28) | 41.1 (40.2 - 42.0) |
| **Pennsylvania** | 36.35 (36.11 - 36.59) | 35.89 (35.27 - 36.52) | 36.1 (35.7 - 36.6) |
| **Rhode Island** | 47.12 (46.15 - 48.09) | 42.27 (39.92 - 44.63) | 44.7 (43.0 - 46.4) |
| **South Carolina** | 35.44 (34.99 - 35.88) | 40.31 (39.20 - 41.42) | 37.9 (37.1 - 38.7) |
| **South Dakota** | 32.78 (31.84 - 33.71) | 46.24 (43.38 - 49.09) | 39.5 (37.6 - 41.4) |
| **Tennessee** | 44.36 (43.94 - 44.79) | 47.08 (46.02 - 48.15) | 45.7 (45.0 - 46.5) |
| **Texas** | 33.39 (33.18 - 33.60) | 36.37 (35.86 - 36.88) | 34.9 (34.5 - 35.2) |
| **Utah** | 21.16 (20.63 - 21.70) | 26.18 (24.78 - 27.58) | 23.7 (22.7 - 24.6) |
| **Vermont** | 49.01 (47.68 - 50.34) | 47.87 (44.66 - 51.07) | 48.4 (46.2 - 50.7) |
| **Virginia** | 25.15 (24.86 - 25.45) | 33.59 (32.79 - 34.40) | 29.4 (28.8 - 29.9) |
| **Washington** | 40.46 (40.06 - 40.85) | 43.03 (42.04 - 44.01) | 41.7 (41.1 - 42.4) |
| **West Virginia** | 45.60 (44.87 - 46.33) | 50.14 (48.16 - 52.12) | 47.9 (46.5 - 49.2) |
| **Wisconsin** | 35.84 (35.46 - 36.22) | 49.17 (48.04 - 50.31) | 42.5 (41.8 - 43.3) |
| **Wyoming** | 30.13 (28.93 - 31.34) | 44.68 (41.12 - 48.24) | 37.4 (35.0 - 39.8) |

**Supplemental Table 7:** Absolute number of deaths stratified by state for ischemic heart disease (IHD) and arrythmias in the United States, 1999–2023.

| **Absolute no. of Deaths** | | | |
| --- | --- | --- | --- |
| **State** | **1999-2020** | **2021-2023** | **1999-2023** |
| **Alabama** | 18395 | 3057 | 21452 |
| **Alaska** | 1449 | 315 | 1764 |
| **Arizona** | 23336 | 6233 | 29569 |
| **Arkansas** | 13419 | 3014 | 16433 |
| **California** | 177119 | 26333 | 203452 |
| **Colorado** | 16779 | 4766 | 21545 |
| **Connecticut** | 17082 | 1907 | 18989 |
| **Delaware** | 6064 | 1089 | 7153 |
| **District of Columbia** | 2198 | 329 | 2527 |
| **Florida** | 99401 | 21632 | 121033 |
| **Georgia** | 24114 | 4315 | 28429 |
| **Hawaii** | 5589 | 910 | 6499 |
| **Idaho** | 7394 | 1707 | 9101 |
| **Illinois** | 54323 | 7553 | 61876 |
| **Indiana** | 37041 | 6464 | 43505 |
| **Iowa** | 20094 | 3468 | 23562 |
| **Kansas** | 14919 | 2719 | 17638 |
| **Kentucky** | 22627 | 4259 | 26886 |
| **Louisiana** | 16572 | 3818 | 20390 |
| **Maine** | 8473 | 1789 | 10262 |
| **Maryland** | 29533 | 5837 | 35370 |
| **Massachusetts** | 30344 | 5059 | 35403 |
| **Michigan** | 52640 | 8327 | 60967 |
| **Minnesota** | 27052 | 6557 | 33609 |
| **Mississippi** | 10144 | 2719 | 12863 |
| **Missouri** | 32162 | 4530 | 36692 |
| **Montana** | 4181 | 987 | 5168 |
| **Nebraska** | 10018 | 2216 | 12234 |
| **Nevada** | 5938 | 1847 | 7785 |
| **New Hampshire** | 7064 | 1209 | 8273 |
| **New Jersey** | 48225 | 6452 | 54677 |
| **New Mexico** | 6722 | 1478 | 8200 |
| **New York** | 90917 | 15356 | 106273 |
| **North Carolina** | 44817 | 8973 | 53790 |
| **North Dakota** | 5069 | 693 | 5762 |
| **Ohio** | 87847 | 13135 | 100982 |
| **Oklahoma** | 20790 | 4153 | 24943 |
| **Oregon** | 20475 | 5326 | 25801 |
| **Pennsylvania** | 87357 | 13031 | 100388 |
| **Rhode Island** | 9332 | 1259 | 10591 |
| **South Carolina** | 24782 | 5292 | 30074 |
| **South Dakota** | 4856 | 1039 | 5895 |
| **Tennessee** | 42399 | 7733 | 50132 |
| **Texas** | 99650 | 19814 | 119464 |
| **Utah** | 6034 | 1376 | 7410 |
| **Vermont** | 5283 | 874 | 6157 |
| **Virginia** | 28389 | 6764 | 35153 |
| **Washington** | 40030 | 7497 | 47527 |
| **West Virginia** | 15157 | 2535 | 17692 |
| **Wisconsin** | 34152 | 7370 | 41522 |
| **Wyoming** | 2435 | 621 | 3056 |

**Supplemental Table 8:** Percentage distribution of place of death for ischemic heart disease (IHD) and arrythmias in the United States, 1999–2023.

| **Place of Death** | **% of Total Deaths** |
| --- | --- |
| **Medical Facility** | 47.23 |
| **Decedent's home** | 24.99 |
| **Hospice facility** | 2.96 |
| **Nursing home/long term care** | 21.07 |
| **Other locations** | 3.75 |

**SUPPLEMENTAL FIGURES**

**Supplementary Figure 1:** State stratified Age-adjusted mortality rates (per 100,000) for ischemic heart disease (IHD) and Arrythmias among U.S. adults aged ≥25, 1999 to 2020.

**Supplementary Figure 2:** State stratified Age-adjusted mortality rates (per 100,000) for ischemic heart disease (IHD) and Arrythmias among U.S. adults aged ≥25, 1999 to 2020.

**Supplementary Figure 3:** Place-of-death trends in the U.S. (1999–2023), highlighting IHD-arrhythmias associated mortality.
